# Impacts of Diagnosis-Intervention Packet Payment Reform on the Behaviour of Inpatient Care in Private Hospitals: Evidence From a Pilot City, China

**DOI:** 10.34172/ijhpm.9545

**Published:** 2026-07-20

**Authors:** Ruoxi Chen, Qiao Yang, Weizhuo Chen, Sisi Zhong, Junyi Chen, Jiamei Qian, Yuxuan Chen, Ting Ye

**Affiliations:** ^1^School of Medicine Health Management, Tongji Medical College, Huazhong University of Science and Technology, Wuhan, China.; ^2^Research Centre for Rural Health Service, Key Research Institute of Humanities & Social Sciences of Hubei Provincial Department of Education, Wuhan, China.; ^3^School of Public Health, Tongji Medical College, Huazhong University of Science and Technology, Wuhan, China.

**Keywords:** Diagnosis-Intervention Packet, Payment Reform, Private Hospitals, Medical Expenditure, Medical Efficiency, Medical Quality

## Abstract

**Background:**

An innovative payment method, the diagnosis-intervention packet (DIP), has been piloted in China since 2020 and was fully implemented in City A in November 2022. However, its specific impact on private hospitals remains inadequately explored. This study aimed to examine how the reform influenced inpatient care behaviours in private hospitals.

**Methods:**

Using a dataset comprising 38 296 inpatient reimbursement records from 2021 to 2023 in a western city in China, 5 private hospitals included in the first batch of pilot institutions were selected as the treatment group, while 3 non-pilot private hospitals constituted the control group. Difference-in-differences (DID) analyses was conducted to estimate policy effects on providers’ inpatient care behaviours across four dimensions: cost control, service-volume adjustment, patient type, and quality risk.

**Results:**

Following the reform, significant changes were observed across several outcome dimensions. Regarding service-volume adjustment, the average length of stay decreased significantly (-1.94; 95% CI: -3.75 to -0.13), and average monthly admissions also declined (-30.36; 95% CI: -59.52 to -1.20). No statistically significant change was observed in patient type. For cost control, the average expenditure per admission decreased significantly by US $670.50 (95% CI: -1149.80 to -191.18). Out-of-pocket (OOP) expenditure per admission also declined by US $118.89 (95% CI: -188.88 to -48.90), although the share of OOP expenditure in total costs increased slightly (0.02; 95% CI: 0.05-4.64). As for quality risk, peri-hospitalisation outpatient visits increased significantly (1.473; 95% CI: 0.252-2.694).

**Conclusion:**

The DIP payment reform in private hospitals contributed to cost containment and process optimisation. However, the structural tension between standardised payment mechanisms and market-oriented hospital operations may generate short-term adjustments that prioritise cost control over quality improvement. Under strict cost-control incentives, hospitals may adopt strategic responses such as shortening inpatient stays and adjusting patient composition, which could pose potential risks to healthcare quality.

## Background

**Key Messages**

**Implications for policy makers**

- The diagnosis-intervention packet (DIP) reform showed effectiveness in controlling medical costs and improving service efficiency in private hospitals, highlighting its potential as a payment innovation.
- Under DIP-based financial incentives, hospitals may adopt strategic responses such as adjusting service structure and patient composition, which warrants careful monitoring for potential risk-avoidance behaviour.
- Particular attention should be paid to potential quality risks arising from the tension between standardised payment protocols and the market-oriented operating model of private hospitals.
- Policy refinement should consider a tiered payment system with differentiated standards and dynamic adjustment mechanisms, together with stronger quality monitoring and more balanced incentives tailored to the characteristics of private hospitals.

**Implications for the public**

China’s ongoing healthcare reforms aim to build a more efficient and sustainable health system. Diagnosis-intervention packet (DIP) is an innovative payment method designed to help hospitals improve resource allocation and control costs. Under this approach, hospitals are encouraged to improve service efficiency while maintaining the quality of care. For patients, the reform may contribute to more standardised and clinically appropriate treatment processes. At the same time, quality monitoring mechanisms are needed to ensure that cost control does not compromise patient outcomes. With continued policy refinement, DIP may help better balance affordability and quality in healthcare delivery.

The diagnosis-intervention packet (DIP) payment reform is an important innovation in China’s health insurance system. It combines case-based payment with a global budget mechanism to improve the allocation of medical resources and contain cost growth.^1^ The DIP reform is grounded in regional medical insurance fund revenue and assigns scores to diagnoses and treatments according to disease groups.^2^ Through total budget control, monthly pre-allocation, and annual reconciliation, it encourages medical institutions to standardise their service practices.^3^ Since the National Medical Insurance Bureau initiated the national implementation of DIP in 2020, the reform has become a key policy tool shaping the medical service delivery system.^4,5^

 DIP exerts its influence through two mechanisms. First, under fixed payment standards, hospitals can retain any surplus if the treatment cost for a DIP-defined group is lower than the reimbursed amount.^6^ Such incentives may encourage hospitals to optimise treatment pathways, shorten length of stay, and reduce unnecessary services.^7^ Conversely, when treatment costs exceed the reimbursement amount, hospitals must absorb the excess cost themselves.^8^ This shift from retrospective to prospective payment reflects a broader international trend in healthcare financing. Similar to diagnosis-related group systems adopted in Organisation for Economic Co-operation and Development countries and several Asian countries such as Japan, South Korea, and Thailand, DIP aims to control costs by shifting incentives from volume-based to value-oriented care.^9^ However, evidence from countries such as Switzerland and France suggests that although such systems can reduce length of stay, they may also induce strategic behaviours, including patient selection, service upcoding, and fragmented hospitalisation.^10,11^ These behaviours may adversely affect access to care and healthcare quality.^12-14^

 Private hospitals have become an indispensable component of China’s healthcare delivery system, particularly in meeting diverse medical needs. By 2022, private hospitals accounted for 21.3% of total outpatient visits in China, and their market share exceeded 40% in specialised fields such as ophthalmology and stomatology. Owing to their market-oriented governance and flexible capital allocation, private hospitals may respond rapidly to payment reforms, but they also face stronger cost-control pressures. Unlike public hospitals, which are generally mission driven and supported by stable government subsidies, private hospitals operate under stronger financial incentives and weaker institutional constraints. Their profit-oriented nature, together with relatively underdeveloped internal governance and quality management systems, may lead to heterogeneous behavioural responses under cost-containment pressure. Regulatory audits have reported a 14.6% non-compliance rate in private hospitals compared with 3.2% in public institutions, with common issues including upcoding and service unbundling. This institutional divergence suggests that private hospitals are not only highly sensitive to DIP reform but may also be major carriers of potential policy risks.

 Existing studies have evaluated the impact of DIP reform mainly in terms of cost containment, service efficiency, and quality of care. Evidence suggests a slower growth rate in average expenditure per hospitalisation and a reduction in patients’ out-of-pocket (OOP) share by approximately 2 to 6 percentage points, which has alleviated some of the financial burden.^15-18^ With respect to service efficiency, a significant reduction in average length of stay has been consistently observed across multiple regions. However, findings on healthcare quality remain mixed. Some studies have reported stable or improved outcomes, including lower in-hospital mortality and reduced 30-day readmission rates.^19^ By contrast, other studies have highlighted potential compromises in care quality associated with strategic behaviours such as disease-group upcoding and service shifting.^20,21^ Despite these advances, several important issues remain unresolved. First, most existing analyses focus on public hospitals, and systematic evidence on how private hospitals respond to institutional pressure is limited. Second, commonly used outcome measures are often restricted to average cost per admission and length of stay, and rarely capture multiple dimensions of hospital behaviour. These gaps limit the ability of policy design to align with actual service delivery and may hinder the full realisation of the intended effects of DIP reform.

 To address these gaps, this study used a difference-in-differences (DID) design to examine the impact of DIP reform on inpatient care behaviour in private hospitals. The outcomes of interest covered four dimensions: cost control, service-volume adjustment, patient type, and quality risk. In addition, we examined whether the effects differed across patient subgroups and whether the reform had potential implications for health equity and healthcare accessibility.

## Methods

 Based on a medical insurance payment reform pilot programme in City A, this study conducted an empirical analysis using a DID design to assess the impact of DIP reform on inpatient service supply behaviours in private hospitals.

###  Data Collection

 The DIP payment reform was formally implemented in City A in November 2022. Accordingly, the period from January 2021 to September 2022 was defined as the pre-implementation period, and the year 2023 was defined as the post-implementation period.

 Our dataset comprised claims reimbursement data from 2021 to 2023 obtained from the Medical Security Bureau. A total of eight comprehensive private hospitals in City A were purposively selected for analysis based on comparable baseline characteristics, including bed capacity, annual inpatient volume, and specialty composition. Among them, five hospitals included in the first batch of DIP pilot institutions were assigned to the treatment group, while three non-pilot private hospitals with similar operational characteristics were selected as the control group.

 During data processing, we restricted admissions and discharges to the calendar interval from January 1 to November 30 of each year to exclude cases spanning calendar years. Core variables retained for analysis included patient identification number, demographic characteristics, cost breakdowns, diagnostic and treatment information, and hospital attributes. After rigorous data cleaning, 2954 records were excluded, accounting for 7.16% of the original sample of 41 250 inpatient records. Exclusions were due to cross-year hospitalisations, missing key variables, logical inconsistencies in length of stay, or non-local reimbursement cases. The final analytical sample comprised 38 296 valid inpatient records.

###  Definition of Variables

 A DID model was used to estimate the policy effect. The primary explanatory variables were a treatment-group dummy variable (treat_i_: 1 for pilot hospitals, 0 otherwise) and a post-implementation time dummy (post_t_: 0 for January 2021–September 2022, 1 for all of 2023).

 The outcome variables in this study captured inpatient service supply behaviours across four dimensions.

1. Service-volume adjustment: This dimension captures changes in the scale and efficiency of service delivery. Length of stay was measured as the number of days between admission and discharge. Average monthly admissions were calculated as the total number of discharges per hospital per month. The admission-to-patient ratio was calculated by dividing the total number of hospitalisations by the number of unique patients, identified by national ID numbers, within each month.
2. Patient type: This dimension evaluates whether hospitals engaged in strategic patient selection. The proportion of mild cases was defined as the percentage of inpatient admissions whose primary diagnosis ICD-10 (International Classification of Diseases, 10th Revision) codes matched the National Health Commission’s low-risk/low-cost catalogue. Similarly, the proportion of short-stay admissions was defined as the percentage of admissions with a length of stay of three days or less.
3. Cost control: This dimension reflects hospitals’ financial responses to fixed payment standards. Average expenditure per admission and OOP expenditure per admission were defined as the arithmetic mean of total medical costs and patient-paid expenses per discharge, respectively. The share of OOP expenditure was calculated as the ratio of individual payments to total hospital charges for each record.
4. Quality risk: This dimension assesses the potential impact of the reform on clinical stability and service integrity. Readmission rates were identified by linking sequential inpatient records for the same individual and measuring the discharge-to-admission interval. Readmission proportions were calculated within specific time intervals (1, 2, 7, 14, 30, 90, and 180 days). In addition, peri-hospitalisation outpatient visits were measured by summing all outpatient encounters for the same patient within a 14-day window, spanning from 7 days before admission to 7 days after discharge.^22^

The control variables included patient-level covariates: gender, age, ethnicity, insurance type, and Charlson comorbidity index.^23,24^

###  Statistical Analysis 

 The DID regression model was specified as follows:


(1) $$Y_{it}=\partial_{0}+\beta_{1}\times post_{t}+\beta_{2}\times treat_{i}+\beta_{3}\times Post_{t}\times treat_{i}+\gamma \times X_{it}+\delta_{i}+\lambda_{t}+\varepsilon_{it}$$

where *Y_it_* denotes the outcome variable for hospital *i* at time *t*. *β_3_* is the key parameter of interest, capturing the net effect of DIP reform. *X_it_* represents the covariates. *δ_i_* and *λ_t_* denote hospital and year fixed effects, respectively. Standard errors were clustered at the hospital level.

 To further assess the robustness of the DID estimates, we applied the least absolute shrinkage and selection operator (LASSO) regression as a regularization-based check. By introducing an L1 penalty term into the objective function, LASSO shrinks less informative coefficients towards zero, which helps mitigate overfitting and potential multicollinearity among covariates. This approach was used to examine whether the core policy effect remained stable under penalised variable selection.

 In addition, subsample regressions were conducted to explore heterogeneity in policy effects across age groups and insurance types.

## Results

 Before estimating the DID model, we tested the parallel trends assumption using an event-study specification. Figure S1 (Supplementary file 1) shows that the estimated coefficients for the pre-reform periods (pre3, pre2, pre1) were not statistically different from zero, and their 95% confidence intervals (CIs) included zero. This indicates that there were no systematic pre-existing differences in outcome trends between the treatment and control groups, supporting the validity of the parallel trends assumption.

Table 1 presents the descriptive statistics of the study sample. In the pre-reform period, pilot hospitals had significantly higher average expenditures and a different patient composition than non-pilot hospitals. For example, patients treated in pilot hospitals were younger, and a larger share of admissions was concentrated in tertiary hospitals (*P*< .001). In the post-reform period, although absolute medical expenditures increased in both groups, the gap between pilot and non-pilot hospitals narrowed.

**Table 1:** Descriptive Statistics of Inpatient Admissions in Pilot and Non-pilot Hospitals Before and After Reform

		**Pre-reform**			**Post-reform**		
		**Non-pilot Hospitals**	**Pilot Hospitals**	***P*** ** Value**	**Non-pilot Hospitals**	**Pilot Hospitals**	***P*** ** Value**
Number of inpatient admissions		1725	18 552		1397	12788	
Medical expenditure, mean (SD)		655.29 (361.92)	1364.32 (2493.06)	<.001	1599.94 (993.01)	1595.30 (2809.70)	.95
OOP, mean (SD)		91.27 (117.82)	249.55 (545.97)	<.001	253.60 (225.48)	284.16 (581.44)	.051
Fund payments, mean (SD)		564.02 (330.70)	1114.77 (2098.14)	<.001	1346.34 (899.64)	1311.15 (2442.61)	.59
Age, mean (SD)		49.18 (16.86)	31.63 (24.86)	<.001	59.82 (14.84)	45.33 (19.62)	<.001
Gender (%)	Male	439 (25.45)	7385 (39.81)	<.001	513 (36.72)	5265 (41.17)	.001
	Female	1286 (74.55)	11 163 (60.19)		884 (63.28)	7523 (58.83)	
	Unknown	0 (0.00)	4 (0.00)		0 (0.00)	0 (0.00)	
Type of medical insurance (%)	UEBMI	332 (19.25)	3972 (21.40)	.44	221 (15.82)	2733 (21.37)	<.001
	URRBMI	1393 (80.75)	14 580 (78.60)		1176 (84.18)	10 055 (78.63)	
Levels of hospitals (%)	Primary	1625 (94.20)	10 371 (55.90)	<.001	1397 (100.00)	5936 (46.42)	<.001
	Secondary	0 (0.00)	944 (5.09)		0 (0.00)	1640 (12.82)	
	Tertiary	100 (5.80)	7237 (39.01)		0 (0.00)	5212 (40.76)	
Ethnicity (%)	Han	1637 (94.90)	16 843 (90.79)	.004	1366 (97.87)	11 779 (92.11)	<.001
	Tibetan	50 (2.90)	1470 (7.92)	.004	22 (1.57)	885 (6.92)	
	Other	38 (2.20)	239 (1.29)		9 (0.64)	124 (0.97)	
1-Day readmission (%)	No	1700 (98.55)	18 375 (99.05)	.59	1396 (99.93)	12 520 (97.90)	<.001
	Yes	25 (1.45)	177 (0.95)		1 (0.07)	268 (2.10)	
2-Day readmission (%)	No	1700 (98.55)	18 363 (98.98)	.68	1396 (99.93)	12 511 (97.83)	<.001
	Yes	25 (1.45)	189 (1.02)		1 (0.07)	277 (2.17)	
1-Week readmission (%)	No	1700 (98.55)	18 317 (98.73)	.996	1394 (99.79)	12 473 (97.54)	<.001
	Yes	25 (1.45)	235 (1.27)		3 (0.21)	315 (2.46)	
2-Week readmission (%)	No	1700 (98.55)	18 227 (98.25)	.89	1388 (99.36)	12 392 (96.90)	<0.001
	Yes	25 (1.45)	325 (1.75)		9 (0.64)	396 (3.10)	
1-Month readmission (%)	No	1700 (98.55)	17 940 (96.70)	.13	1368 (97.92)	12 005 (93.88)	<.001
	Yes	25 (1.45)	612 (3.30)		29 (2.08)	783 (6.12)	
3-Month readmission (%)	No	1700 (98.55)	17 634 (95.05)	.012	1333 (95.42)	11 687 (91.39)	<.001
	Yes	25 (1.45)	918 (4.95)		64 (4.58)	1101 (8.61)	
6-Month readmission (%)	No	1687 (97.80)	17 481 (94.23)	.016	1317 (94.27)	11 565 (90.44)	<.001
	Yes	38 (2.20)	1071 (5.77)		80 (5.73)	1223 (9.56)	
Referral (%)	None	1524 (88.35)	15 320 (82.58)	.017	1063 (76.09)	9016 (70.50)	<.001
	Upward	0 (0.00)	178 (0.96)		0 (0.00)	120 (0.94)	
	Lateral	176 (10.20)	2899 (15.63)		318 (22.76)	3578 (27.98)	
	Downward	25 (1.40)	155 (0.84)		16 (1.15)	74 (0.58)	

Abbreviations: OOP, out-of-pocket; SD, standard deviation; UEBMI, the Urban Employee Basic Medical Insurance; URRBMI, Urban and Rural Resident Basic Medical Insurance.

 The DID estimates for the impact of DIP implementation are summarised in Tables 2 and 3. Regarding service-volume adjustment, the average length of stay decreased significantly by 1.94 days (*P*= .04), while average monthly admissions declined by 30.36 cases (*P*= .05). The admission-to-patient ratio increased by 54.1%, although this change did not reach conventional statistical significance (*P*= .08). In terms of patient type, no significant change was observed in the proportion of mild-case admissions (*P*= .41), whereas the proportion of short-stay admissions declined by 19.8 percentage points and was marginally significant (*P*= .067). For cost control, the DIP reform was associated with significant reductions in average expenditure and OOP expenditure per admission of US $670.49 (*P*= .010) and US $118.89 (*P*= .003), respectively, although the share of OOP expenditure increased significantly by 2.3 percentage points (*P*= .046). For quality risk, the coefficients for readmission rates at 1, 2, 7, 14, 30, 90, and 180 days were positive but not statistically significant, with *P* values ranging from 0.079 to 0.185. By contrast, outpatient visits during the peri-hospitalisation period increased significantly by 1.47 visits (*P*= .033).

**Table 2:** Effect of Diagnosis-Intervention Packet Implementation on All Continuous Outcomes

**Variables**	**Estimate**	**95% CI**	**t**	**Pre-reform mean**	**% Change**
Service efficiency					
Length of stay	-1.94*	(-3.75, -0.13)	-2.10	8.68	-22.3%
Average monthly admissions	-30.36*	(-59.52, -1.20)	-2.04	176.70	-17.2%
Admission-to-patient ratio	0.73	(-0.03, 1.48)	1.91	1.34	+54.1%
Cost control					
Average expenditure per hospital admission	-670.49**	(-1149.80, -191.18)	-3.00	1364.32	-49.1%
OOP per hospital admission	-118.89**	(-188.88, -48.90)	-3.64	249.55	-47.6%
Quality risk					
Outpatient visits during peri-hospitalisation period	1.47*	(0.25, 2.69)	2.36	0.38	+387.6%

Abbreviations: OOP, out-of-pocket; CI, confidence interval. *Notes*: Asterisks denote statistical significance: * *P* < .05, ** *P* < .01. All models adjusted for patient-level covariates (age, gender, ethnicity, insurance type, hospital level, and Charlson comorbidity index) and included year and hospital fixed effects.

**Table 3:** Effects of Diagnosis-Intervention Packet Implementation on Proportion and Binary Outcomes

**Variables**	**Estimate**	**95% CI**	**t**	**Pre-reform Mean**	**Percentage-Point Change**
Patient type					
Proportion of mild-case	-0.07	(-0.22, 0.09)	-0.84	32.94%	-6.5 pp
Proportion of short-stay admissions	-0.20	(-0.41, 0.01)	-1.83	24.08%	-19.8 pp
Cost control					
Proportion of OOP expenditure	0.02*	(0.00, 0.05)	2.09	18.29%	+2.3 pp
Quality risk					
1-Day readmission	0.11	(-0.00, 0.22)	1.89	0.97%	+11.0 pp
2-Day readmission	0.11	(-0.01, 0.22)	1.83	1.01%	+11.1 pp
1-Week readmission	0.11	(-0.01, 0.23)	1.89	1.21%	+10.9 pp
2-Week readmission	0.11	(-0.01, 0.23)	1.83	1.75%	+10.8 pp
1-Month readmission	0.10	(-0.02, 0.21)	1.74	3.32%	+9.6 pp
3-Month readmission	0.08	(-0.02, 0.18)	1.64	4.58%	+8.3 pp
6-Month readmission	0.07	(-0.03, 0.18)	1.39	5.18%	+7.3 pp

Abbreviations: OOP, out-of-pocket; CI, confidence interval; pp, percentage points. *Notes*: Asterisks denote statistical significance: * *P* < .05. All models adjusted for patient-level covariates (age, gender, ethnicity, insurance type, hospital level, and Charlson comorbidity index) and included year and hospital fixed effects.

 To ensure the reliability of our findings, we first performed a robustness check using LASSO regression, with the estimates remaining broadly consistent with the primary DID results in both direction and magnitude. Detailed LASSO regression results are provided in Table S1 (Supplementary file 1). Besides, we conducted heterogeneity analyses to gain additional insights into the differential policy impacts across demographic and insurance subgroups. The results in Table 4 revealed that the cost-containment effects were more pronounced among elderly patients and the Urban and Rural Resident Basic Medical Insurance (URRBMI) enrollees, while notable variations were observed in the share of OOP and length of stay across different insurance types. These heterogeneous patterns provide an empirical basis for understanding the nuanced responses of private hospitals to the DIP reform.

**Table 4:** Estimated Results From a Grouped Regression Analysis by Age and Health Insurance Type

**Variable**	**Group**	**Estimate**	**SE**	***P*** ** Value**	**Sample Size**	**Pre-reform Mean**	**Effect Size**
Average expenditure per hospital admission	Elderly group	-520.74	204.16	0.023	17 644	1350.35	-38.6%
	Young group	-251.48	209.96	0.25	18 652	1129.42	-22.3%
	URRBMI	-624.39	226.24	0.015	28 365	1285.87	-48.60%
	UEBMI	-682.18	296.45	0.04	7931	1072.59	-64.00%
OOP per hospital admission	Elderly group	-94.27	32.64	0.012	17 644	237.42	-39.7%
	Young group	-139.68	56.17	0.026	18 652	215.27	-64.9%
	URRBMI	-128.71	38.66	0.005	28 365	262.52	-49.0%
	UEBMI	-51.00	27.11	0.081	7931	100.91	-50.5%
The share of OOP	Elderly group	0.02	0.02	0.23	17 644	0.14	+1.7 pp
	Young group	-0.00	0.06	0.87	18 652	0.15	-0.2 pp
	URRBMI	0.01	0.01	0.54	28 365	0.17	+0.7 pp
	UEBMI	0.10	0.02	<0.001	7931	0.09	+9.9 pp
Length of stay	Elderly group	7.06	1.78	0.001	17 644	9.16	+77.1%
	Young group	2.60	0.95	0.016	18 652	8.23	+31.6%
	URRBMI	6.76	1.79	0.002	28 365	8.60	+78.5%
	UEBMI	4.08	1.15	0.003	7931	8.95	+45.6%

Abbreviations: OOP, out-of-pocket; SE, standard error; UEBMI, Urban Employee Basic Medical Insurance; URRBMI, Urban and Rural Resident Basic Medical Insurance; pp, percentage points.

## Discussion

 Using inpatient claims data from 2021 to 2023, this study conducted an empirical analysis of service provision in pilot and non-pilot private hospitals. The findings indicated that the DIP reform achieved its intended objectives of cost containment and process optimisation in private hospitals. However, under strong cost-control pressures, hospitals also responded strategically by compressing inpatient services or adjusting patient composition, which suggested potential risks to care quality.

 In terms of service-volume adjustment, private hospitals experienced shorter inpatient stays and higher resource turnover during the reform period, while overall admission volume declined. On the one hand, the reduction in length of stay improved efficiency, which helped lower total expenditures and increase hospital surpluses to some extent. This finding was consistent with the results reported by Ding and Cai.^25^ On the other hand, the decline in average monthly admissions together with the increase in the admission-to-patient ratio suggested that hospitals may have applied stricter admission criteria, thereby reducing unnecessary hospitalisations. Meanwhile, studies from other regions in China, such as Jiangxi and Guangdong, reported opposite trends, with significant increases in admissions per capita, total inpatient numbers, and overall admission volume.^16,26^ These differences in findings across studies may have been attributable to variations in reform context, study population, and research design.^27,28^ Taken together, these results suggested that the DIP reform placed hospitals in a complex environment of competing constraints and incentives, leading to dynamic strategic adjustments.

 Regarding patient type, neither the proportion of mild-case admissions nor the proportion of short-stay admissions reached conventional levels of statistical significance. The non-significant mild-case indicator suggested that hospitals did not markedly alter their case composition by admitting a larger share of mild cases after the reform. Although the short-stay indicator did not reach conventional statistical significance, its direction was negative and its magnitude was economically meaningful; therefore, residual case-mix adjustment could not be fully ruled out. Overall, these findings suggest that compositional changes were limited and did not represent a substantial shift towards a milder or shorter-stay case mix. Importantly, the simultaneous decline in average length of stay and short-stay admissions was consistent with hospitals compressing inpatient care and shifting some short-stay demand to outpatient or day-surgery settings. In other words, the observed changes appeared more likely to reflect service-path reorganisation and delivery adjustment than straightforward selection of mild cases. Taken together, these findings partially alleviate concerns that case-mix changes might have driven the observed per-admission cost and quality outcomes, but they do not eliminate those concerns entirely. Accordingly, the subsequent cost and quality results were interpreted cautiously as reduced-form effects that may reflect both direct payment-reform effects and potential changes in patient composition.

 For cost control, the DIP reform played a significant role in curbing the growth of inpatient expenditures and reducing patients’ direct financial burden. We found that the growth rate of inpatient costs in pilot hospitals was significantly lower than that in the control group, indicating that the DIP payment mechanism was effective in restraining the expansion of healthcare costs. This trend was consistent with evidence reported in Suqian and Nanchang.^15,16^ Taken together with the patient-type results, the fact that the proportion of mild-case admissions did not change significantly and the proportion of short-stay admissions declined only marginally suggests that, although the reduction in per-admission expenditure primarily reflected the direct cost-containment effect of the payment reform, some influence from changes in patient composition could not be entirely excluded. In other words, the decline in expenditure was likely the result of a combination of payment constraints, service-process compression, and modest case-mix adjustment, rather than a single mechanism. Importantly, the main models had already adjusted for age, insurance type, ethnicity, Charlson comorbidity index, as well as hospital and year fixed effects, which helped mitigate confounding from observable severity differences. Besides, this study also showed that although overall cost growth was contained, the proportion of OOP expenses borne by patients increased moderately. By contrast, Chen et al reported a significant decline in OOP ratios after the reform in Huai’an and Nanchang, which differed from the findings of this study.^29^ Such discrepancies may have been attributable to regional differences in medical insurance reimbursement rates, or to differences in cost-shifting mechanisms between private and public hospitals. In addition, differences in disease-weighting settings across regions, together with the introduction of additional non-reimbursable services by some hospitals to offset revenue loss, may also have contributed to the observed changes.^30^ These findings suggested that, while costs were being contained, the medical insurance payment structure was undergoing a certain rebalancing, and the transmission effects of DIP reform were highly complex.

 As for quality risk, hospitals may have responded to DIP constraints by transferring certain inpatient service components to outpatient settings or by adopting strategies such as fragmented hospitalisation. The results showed a significant increase in peri-hospitalisation outpatient visits, together with a marginal rise in short-term readmission rates and a marked reduction in average length of stay. Taken together with the patient-type findings, these patterns suggest that inpatient service pathways had been reorganised under DIP constraints rather than simply selecting more short-stay cases. Specifically, the increase in peri-hospitalisation outpatient visits and the rise in readmission risk may reflect behavioural responses to DIP incentives, but they may also be partly attributable to service shifting and compression of inpatient processes. Nevertheless, these patterns still point to possible fragmentation of hospital care and potential compromises in the adequacy of diagnosis and treatment, which could pose risks to care quality. This interpretation is consistent with Alexander Kutz’s study of the Swiss diagnosis-related group system.^10^ By contrast, studies from France and from the Chinese cities of Beijing and Zhongshan found no significant change in readmission rates after payment reform, whereas research from Korea reported a significant decline.^11,26,31,32^ A possible explanation was that private hospitals, driven by stronger profit motives, may have been more willing to trade off quality for short-term cost savings, making external regulation more important than internal incentives for quality assurance.

 Finally, our heterogeneity analysis revealed pronounced differences across age groups and insurance types, raising important equity considerations.^33^ The larger cost reductions observed among older patients and URRBMI inpatients, who typically have greater healthcare needs and lower income, suggested that the reform may have enhanced financial protection for vulnerable populations, partly because of their more inelastic demand for services and higher reimbursement rates.^34,35^ In contrast, younger patients and the Urban Employee Basic Medical Insurance (UEBMI) inpatients showed weaker responses to the policy, indicating conditional heterogeneity in policy effects across subpopulations. Furthermore, under pressure from DIP reform, private hospitals were driven by both economic incentives and risk aversion. While hospitals may have achieved short-term efficiency gains through cost control and process redesign, they may also have adopted strategies to avoid patients with complex conditions or high treatment costs, thereby exacerbating inequities in access to and quality of care for marginalised groups. These behaviours highlighted the sharp conflict between efficiency-oriented reform and equity goals, and they suggested that without explicit equity safeguards, payment reforms may fail to protect vulnerable populations and may even harm their interests.

 This study has several strengths. First, the data included 38 296 inpatient reimbursement records from the Medical Security Bureau in City A, ensuring adequate data quality and sample size for evaluation. Second, we constructed a multidimensional outcome indicator system covering cost control, service-volume adjustment, patient type, and quality risk, which enabled a more comprehensive assessment of the impact of DIP reform on hospital behaviours across multiple dimensions. Third, the use of the DID design strengthened the internal validity of our causal inference regarding the effects of DIP reform. Finally, we stratified hospitalised patients by age group and insurance type to examine whether DIP implementation produced differential effects across populations, with the aim of exploring potential disparities in healthcare equity.

 This study also has several limitations. First, the sample was drawn solely from selected pilot and control private hospitals in a single city, which may limit the external validity of the findings. Furthermore, the absence of comparable inpatient claims data for local public hospitals prevented a direct one-to-one comparison, making it difficult to fully disentangle ownership-specific effects from city-level heterogeneity. Second, the relatively short observation window limited our ability to capture the long-term effects of DIP reform. Nevertheless, as a national pilot programme, DIP can still provide valuable insights for other regions planning to implement the policy. Finally, although subgroup analyses were conducted across different populations, unobserved heterogeneity may still exist within each subgroup.

## Conclusions

 Using municipal insurance claims data, this study empirically evaluated the impact of DIP reform on inpatient service behaviours in private hospitals and examined the underlying mechanisms. The results indicate that the reform partially achieved its objectives of cost control and process optimisation. However, the tension between standardised payment protocols and market-oriented hospital operations has created a short-term equilibrium in which cost reduction is prioritised over quality improvement. Future studies should focus on evaluating the long-term implications of the reform for care quality, financial sustainability, operational efficiency, and health equity, to support the refinement of health insurance payment policies and facilitate the sustainable development of private hospitals.

## Acknowledgements

 We thank the Medical Security Bureau in City A for providing historical data in the study, and those who reviewed drafts of this paper.

## Disclosure of artificial intelligence (AI) use

 Sections of manuscript affected: Results and Discussion.

 Type of AI tool used: ChatGPT.

 Purpose of use: Language editing.

## Ethical issues

 Ethics approval for this study was granted by the Medical Ethics Committee of Tongji Medical College, Huazhong University of Science and Technology (protocol numbers: [2022](S092)). The study was performed in accordance with the Declaration of Helsinki. The manuscript does not contain any personally identifiable data in any form.

## Conflicts of interest

 Authors declare that they have no conflicts of interest.

## Disclaimer

 The opinions expressed in this paper are those of the authors alone and do not necessarily reflect the official policy or position of *International Journal of Health Policy and Management*, its editors, or any affiliated institutions.

## Supplementary files


Supplementary file 1 contains Figure S1 and Table S1.
