# Supplementary material for: Impacts of Diagnosis-Intervention Packet Payment Reform on the Behaviour of Inpatient Care in Private Hospitals: Evidence From a Pilot City, China
Source: Int J Health Policy Manag. 2026 Jul 20;15:9545. doi: 10.34172/ijhpm.9545 (PMC13770218; doi:10.34172/ijhpm.9545)
Supplement: Supplementary file 1 [file ijhpm-15-9545-s001.pdf]

Article title: Impacts of Diagnosis-Intervention Packet Payment Reform on the Behaviour of Inpatient Care in Private Hospitals: Evidence From a Pilot City, China

Journal name: International Journal of Health Policy and Management (IJHPM)

Authors' information: Ruoxi Chen[1,2], Qiao Yang[1,2], Weizhuo Chen[1,2], Sisi Zhong[1,2], Junyi Chen[1,2], Jiamei Qian[1,2], Yuxuan Chen[3], Ting Ye[1,2]*

[1]School of Medicine Health Management, Tongji Medical College, Huazhong University of Science and Technology, Wuhan, China.

[2]Research Centre for Rural Health Service, Key Research Institute of Humanities & Social Sciences of Hubei Provincial Department of Education, Wuhan, China.

[3]School of Public Health, Tongji Medical College, Huazhong University of Science and Technology, Wuhan, China

***Correspondence to:** Ting Ye; Email: yeting@hust.edu.cn

Citation: Chen R, Yang Q, Chen W, et al. Impacts of diagnosis-intervention packet payment reform on the behaviour of inpatient care in private hospitals: evidence from a pilot city, China. Int J Health Policy Manag.2026;15:9545. doi:10.34172/ijhpm.9545

Supplementary file 1

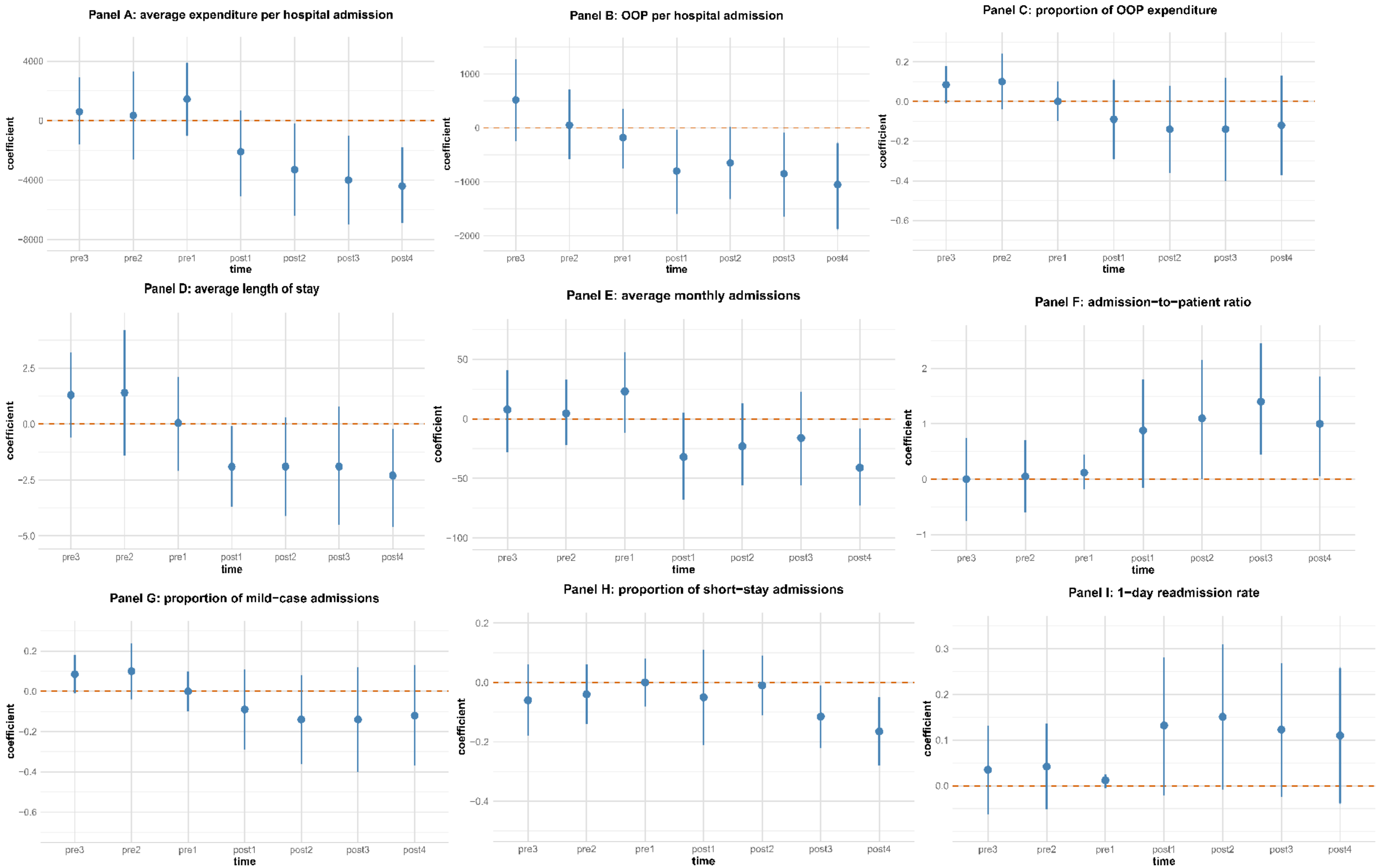
Panel A: average expenditure per hospital admission
4000
0
−4000
−8000
coefficient
pre3
pre2
pre1
post1
post2
post3
post4
time
Panel B: OOP per hospital admission
1000
0
−1000
−2000
coefficient
pre3
pre2
pre1
post1
post2
post3
post4
time
Panel C: proportion of OOP expenditure
0.2
0.0
−0.2
−0.4
−0.6
coefficient
pre3
pre2
pre1
post1
post2
post3
post4
time
Panel D: average length of stay
2.5
0.0
−2.5
−5.0
coefficient
pre3
pre2
pre1
post1
post2
post3
post4
time
Panel E: average monthly admissions
50
0
−50
−100
coefficient
pre3
pre2
pre1
post1
post2
post3
post4
time
Panel F: admission−to−patient ratio
2
1
0
−1
coefficient
pre3
pre2
pre1
post1
post2
post3
post4
time
Panel G: proportion of mild−case admissions
0.2
0.0
−0.2
−0.4
−0.6
coefficient
pre3
pre2
pre1
post1
post2
post3
post4
time
Panel H: proportion of short−stay admissions
0.2
0.0
−0.2
−0.4
coefficient
pre3
pre2
pre1
post1
post2
post3
post4
time
Panel I: 1−day readmission rate
0.3
0.2
0.1
0.0
coefficient
pre3
pre2
pre1
post1
post2
post3
post4
time

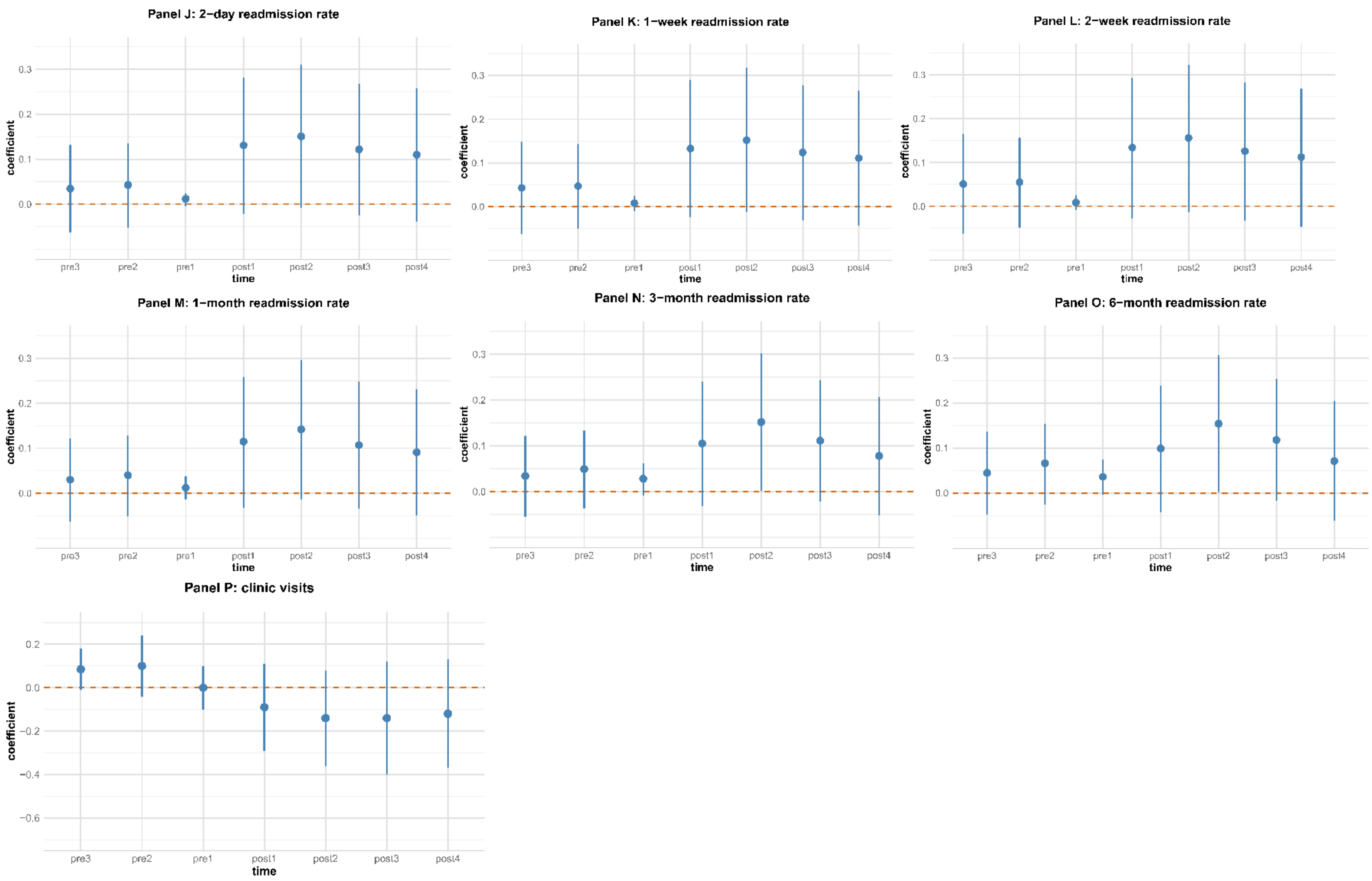

Figure S1. Tests on the validity of parallel assumptions for the whole sample.

Table S1. Penalized regression coefficients from LASSO robustness analysis

	Hospitalization expenditure	OOP per hospital admission
treat*post	-580.26	-94.70
treat	321.40	60.77
post	829.00	138.97
age	2.95	-0.19
Gender(male)	177.41	48.45
Gender(other)	-388.97	-9.49
Ethnicity(minor)	255.42	27.77
Ethnicity(other)	-70.87	-8.80
Insurance type(URRBMI)	153.68	176.16
Hospitals levels (Secondary)	933.74	211.97
Hospitals levels (Tertiary)	430.56	109.06
Charlson	565.39	96.39
intercept term	136.79	-71.13

Notes The table reports penalized coefficient estimates from LASSO regression. Standard errors and conventional significance tests are not reported because LASSO is used here as a regularization-based robustness check rather than as a conventional inferential model.
